# In vitro antiplasmodial activity and prophylactic potentials of extract and fractions of *Trema orientalis* (Linn.) stem bark

**DOI:** 10.1186/s12906-017-1914-x

**Published:** 2017-08-15

**Authors:** John Oludele Olanlokun, Oluwole Moses David, Anthony Jide Afolayan

**Affiliations:** 1 Laboratories for Biomembrane Research and Biotechnology, Department of Biochemistry, Faculty of Basic Medical Sciences, College of Medicine, University of Ibadan, Ibadan, Nigeria; 2 Department of Microbiology, Faculty of Science, Ekiti State University, Ado-Ekiti, Nigeria; 3 Medicinal Plants and Economic Development Research Centre, Botany Department, University of Fort Hare, Alice, 5700 South Africa

**Keywords:** Plasmodium bergei, Hemin, Hemozoin, Ferriprotoporphyrin (ix), *Trema orientalis*, Chloroquine, Sulfadoxine-pyrimetamine

## Abstract

**Background:**

*Trema orientalis* (*T. orientalis* Linn) has been used in the management of malaria in the western part of Nigeria and despite its application in ethnomedicine, there is dearth of scientific evidence to justify the acclaimed prophylactic antimalarial usage of the plant. The aim of this study is to assess the in vitro antiplasmodial cell-free assay and chemopreventive efficacy of the methanol extract of the stem bark of *T. orientalis* and its fractions as a prophylactic regimen for malaria prevention. Also, the antimicrobial activities of the extract and the fractions were investigated.

**Method:**

Vacuum liquid chromatography was used to obtain dichloromethane, ethylacetate and methanol fractions from the methanol extract of *T. orientalis*. The fractions were tested for their prophylactic and cell-free antimalarial activity using murine models and β-hematin formation assay respectively. Disc diffusion method was used to determine the antibacterial activity of the extract and its fractions against both Gram-positive and Gram-negative bacteria.

**Results:**

In the prophylactic experiment, dichloromethane (DCMF), methanol fraction (MF) and extract (ME) (in this order) showed significant chemopreventive effects against *P. berghei* invasion of the red blood cells when compared with both Sulfadoxine-Pyrimethamine (SP) and untreated controls. Results of the in vitro study showed that the DCMF had the highest effect in preventing the formation of β-hematin when compared with other fractions. The DCMF also had the highest percentage inhibition of β-hematin formation when compared with chloroquine. The extract and fractions showed a concentration dependent antibacterial activity. Methanol extract had a pronounced inhibitory effect on *Enterobacter cloaca* ATCC 13047 and *Enterococcus faecalis* ATCC 29212. *Serratia mercescens* ATCC 9986 and *Pseudomonas aeruginosa* ATCC 19582 were the most susceptible bacteria.

**Conclusion:**

The results obtained showed that both extract and fractions of *T. orientalis* possessed antiplasmodial and antimicrobial activity.

## Background

Malaria is a febrile, mosquito-borne infection, which is responsible for symptoms like periodic chills, rigors, profuse sweating and high fevers, which occur at regular intervals of 48 to 72 h [1]. Malaria is a serious and often fatal disease caused by plasmodium parasite that is transfected when mosquito feeds on blood meal from humans. Despite the fact that malaria is a deadly disease, illness and death from malaria can usually be prevented [2]. The geographical distribution of malaria is determined by the distribution of the Anopheles mosquitoes, as well as areas having the correct climate. The transmission has traditionally remained at altitudes below 2000 m [3]. Recently, there has been increased malarial transmission in areas of higher altitudes in Africa, possibly due to the climatic change resulting in increased temperatures in high-altitude areas [4].

The incidence of severe malaria is very high among children below 5 years of age in malaria endemic areas. Patients with severe *P. falciparum* infections often have high parasite counts, and may develop severe symptoms from several organ systems [5].

Malaria’s severity is noticed in children within a short time and this can degenerate further in hours with clinical features such as anaemia, cerebral malaria, hypoglycaemia, jaundice and respiratory distress [6]. Other clinical symptoms such as Kidney failure, metabolic acidosis and high lactate levels have also been reported in severe malaria [7].

Cerebral malaria is one of the complications where the patient has reduced consciousness that can develop into coma or death. Clinical manifestations in relation to this include convulsions, reduced response to painful stimuli, abnormal motor posturing and increased intracranial pressure [8]. Severe hemolytic anaemia has also been associated with malaria infection [9]. This is so because erythrocytes are destroyed coupled with its minimal synthesis, depletion of folate and reduced red blood cell in circulation. The development of anaemia may be gradual or rapid and it may be chronic especially when the infections are persistent [10]. Malaria infection causes reduced blood glucose and this is responsible for high mortality rates among children and this, coupled with anemia and cerebral malaria, increase mortality rate [6]. It is not only *P. falciparum* specie that is responsible for most of the mortality from severe malaria, other species may progress in severity as well. For example, malaria caused by *P. vivax* is often responsible for respiratory symptoms, which may progress to coma. Malaria caused by *P. vivax* can also result in renal failure [11]. *P. vivax* has hypnozoites, which cause relapse of infection. These relapses make the chronic complications to malaria infections, such as anaemia, more severe.

Malaria parasites undergo a series of morphological transformations during their life cycle. Upon invasion of the human host, the parasites invades the liver; undergo maturation before being released into the bloodstream, starting yet another stage called the intraerythrocytic stage. During this stage, the malaria parasite multiplies and changes forms into what looks like a ring called the “ring stage” [12]. At this stage, the parasite degrades hemoglobin for its biosynthetic requirements, releasing free heme also known as Ferriprotoporphyrin (IX) (FePPIX) in the process [13]. Ferriprotoporphyrin (IX) is highly reactive and toxic to plasmodium which if allowed to accumulate, will cause the generation of Reactive Oxygen Species (ROS) which may induce oxidative stress leading to parasitic death [14]. To avoid the toxic effects of heme, trophozoite stage parasites polymerize these molecules within their food vacuole of an estimated pH between 4.5 to 5.0, into a non-toxic, un-reactive, insoluble crystalline compound called *Hemozoin* or “Malaria pigment”. Hemozoin formation thus is considered an important target in the search and finding of new antimalarial drugs [15, 16]. To mimic the exact environments and for further studies on hemozoin in vitro, a synthetic polymer structure made from Ferriprotoporphyrin (IX) named β-hematin is believed to be a structural analog of hemozoin, making it an excellent alternative for the purpose [17, 18].

In spite of several efforts to combat malaria through chemotherapy, vaccines or chemoprevention, the disease remain unabated simply because of the distribution, widespread and survival of the mosquitoes that carry the parasite and multidrug resistance of the parasite to different drugs designed to cure the disease. Again, variability in the developmental stages of the parasite has also made the treatment quite laborious simply because drugs designed for asexual stage of the parasite may not be therapeutically effective against the sexual stages. In Africa for example, poverty, strict adherence to treatment regimen to avoid resistance and exposure to mosquito are the major challenges faced in the eradication of the disease from the continent. As long as the infection and resistance to drugs continue unabated, search for total cure for the disease must also continue to completely eradicate malaria from the continent.

Several approaches had been developed in the treatment of the disease in Africa. One of the most prominent means is the folkloric approach using the herbs with plausible cure for the disease. Decoction prepared from these herbs is either taken for therapeutic purpose or for prevention. *Trema orientalis* Linn Blume is one of such plant with promising antiplasmodial properties. It is a perennial tree that belongs to the family Ulmaceae. Almost every part of the plant is used for traditional medicine in Africa [19]. The stem bark decoction is used as vermifuge and anti-dysenteries [20] while the leaves are mixed with leaves of *Bidens pilosa, Citrus aurantifolia*, and peels of unripe pineapple to treat jaundice, bronchitis, pneumonia and pleurisy [21], the bark and leaf decoction has been reported to have antiplasmodial activities [22]. In Nigeria, the stem bark is peeled and soaked in water or soaked with other plant for drinking and bathing to treat malaria [23, 24]. Our previous experiment on the curative effect of this plant further validated the therapeutic efficacy of the plant [31] and also necessitated our quest for the chemopreventive effect of this same plant extract against malarial infection. Other authors have established the various drug potential [25] and specifically, curative effects of this plant extracts on plasmodium malaria [26]. A previous study had reported that *T. orientalis* had in vitro antiparasitic activity [27]. The animal model (mice) was used in this research because the plasmodium strain (*P. berghei*) develops in the mouse model and because they are lower mammals, possible extrapolation to human biology may be possible to some extent. If truly effective and without prominent side effects, drug candidates can be purified and optimized from this herb and by so doing, complete cure and prevention for the disease is possible. Premised on this fact, we investigated the in vivo chemopreventive effects and the in vitro antiplasmodial activity of the stem bark extract and fraction of *T. orientalis* on *P. berghei*-induced malaria in mice.

## Methods

### Plant source, identification and preparation of solvent extract and fractions

The plant was identified and authenticated in their fresh state by Mr. Omotayo, F.O., the present curate of the Herbarium Unit of Plant Science Department of the Faculty of Science, Ekiti State University, Nigeria. A specimen is domiciled in the Unit and registered with the Identification Number UHAE 2015/35. The stem bark peels of *T. orientalis* were obtained from Ado-Ekiti, Nigeria in March, 2016. The collection site was wild and as a result, did not require authorization from anybody. The stem bark peels were air-dried at room temperature to avoid possible degradation or denaturation of their active compounds. The air-dried stem bark was blended to powder using an electric blender. This was stored in a glass container. Thereafter, 5 kg of blended air-dried stem bark was soaked in 10 L of methanol for 72 h at room temperature. It was continually stirred after each 24 h. After 72 h, the mixture was filtered and the filtrate was concentrated using rotary evaporator under reduced pressure at 40 °C. The crude methanol extract concentrate weighed 53 g.

### Vacuum liquid chromatography purification procedure

The crude methanol extract was further purified using the Vacuum Liquid Chromatography (VLC). The pre-washed sintered glass Buchner was further cleaned with concentrated H_2_SO_4_ to remove impurities from the sieve. The column was then packed three-quarter full with silica gel 60 (0.040–0.063 mm, MERCK). The column was then placed on a conical flask Buchner and connected to the vacuum pump. The crude methanol extract (20 g) was adsorbed to 20 g of the Thin Layer Chromatography gel (particle size 0.040–0.063 mm). The column was packed with n-hexane and the adsorbed sample was applied to the column, the pump was switched on and adsorbed sample was washed first with n-hexane solvent, topically applied to the column, then successively with dichloromethane, ethylacetate and finally with methanol. The fractions obtained were concentrated at 40 °C using rotary evaporator and transferred into pre-weighed all-glass sample bottles with rubber stopper and labeled.

### Prophylaxis experiment

Thirty male Swiss albino mice (15 g) were obtained from the animal house section of the Institute of Advanced Medical Research And Training (IAMRAT), College of Medicine, University of Ibadan, grouped into six groups of five animals each and were acclimatized for two weeks with regular feeding with rat chow and clean water. The animals were kept in clean cages with wooden shaves as beddings in the animal house under 12 dark/light cycle. The animals were randomly assigned into the groups; four experimental groups of five animals each for the test drugs and two control groups of five animals each. The negative control group receive vehicle only while the positive control group received standard drug only.

After acclimatization, the animals were orally pretreated once daily with a daily dose of 50, 100 and 200 mg per kg body weight of methanol extract (ME), methanol (MF), dichloromethane (DCMF), and ethyl acetate (EF) fractions, vehicle and 10 mg per kg body weight of Sulfadoxine-pyrimethamine (SP) which is the standard drug for 1 week before they were transfected with infected erythrocytes from a donor mouse using an inoculums size of 10^7^ of the parasite load of chloroquine-sensitive strain of *P. berghei.* The negative control group animals were treated with the vehicle (5% Dimethylsufoxide (DMSO). Treatment of all the test and control groups were done daily (orally), continued throughout the experiment and thin film slides were collected once in 2 days until day seven of the experiment. Microscopy was carried out with the aid of binocular microscope (Olympus brand) using oil immersion objective. The percentage parasitemia and percentage clearance were used to assess the antiplasmodial efficacy of the drug candidates.

After the seventh day, blood was collected from the animal and were used for the assessment of the Packed Cell Volume (PCV), White Blood Cell (WBC) count and Full Blood Count (FBC).

Percentage Parasitemia and percentage parasite clearance where calculated as follows:

$$ Percentage Parasitemia= Number of infected\  Red\  Blood Cells\frac{counted}{Total} Number of\  Red\  Blood cellc counted\ X\ 100 $$

$$ Percentage Clearance=\frac{Control- Test}{Control}X\ 100 $$

### In vitro antiplasmodial cell-free assay

Antimalarial activity of *T. orientalis* extract and fractions was evaluated by the method described by Afshar et al. [28] with some modifications. Briefly, varying concentrations (10–80 mg/mL freshly dissolved in dimethylsulphoxide (DMSO)) of the extracts and fractions were produced. The reaction mixtures were incubated with 50 μL of 60 mM hemin chloride, 250 μL of 40 mM oleic acid, and 250 μL of 4 M HCl. The final volume was adjusted to 1.0 mL volume using varied volumes of sodium acetate buffer, pH 5, for eighteen h at 37 °C in a shaker water bath. Artesunate and Chloroquine diphosphate were used as positive controls. After incubation, the samples were centrifuged (14,000 rpm, 10 min, at 21 °C) and the hemozoin pellet was repeatedly washed with incubation (15 min at 37 °C with regular shaking) in 2.5% (*w*/*v*) SDS in phosphate buffered saline followed by a final wash in 0.1 M sodium bicarbonate until the supernatant was clear (usually 3–8 washes). After the final wash, the supernatant was discarded and the pellets were dissolved in 1.0 mL of 0.1 M NaOH before determining the hemozoin content by measuring the absorbance at 400 nm (752 N BOSCH UV-VIS spectrophotometer). The results were recorded as % inhibition (*I*%) of heme crystallization compared to negative control (DMSO) using the following equation:

$$ I\%=\left[\left( AN- AS\right)/ AN\right]x\ 100 $$

where, *AN* is absorbance of negative control and *AS* is absorbance of test samples.

### Antibacterial activity of the extract and fractions of *T. orientalis*

The bacteria used in this study were collected from the Department of Microbiology Laboratory, Ekiti State University, Ado-Ekiti, Nigeria. The organisms include four Gram negative bacteria (*Enterobacter cloaca* ATCC 13047, *Escherichia coli* ATCC 8739, *Serratia mercescens* ATCC 9986 and *Pseudomonas aeruginosa* ATCC 19582) and two Gram positive bacteria (*Enterococcus faecalis* ATCC 29212 and *Staphylococcus aureus* ATCC 6538). The test bacteria were grown at 37 °C in Mueller-Hilton broth (Oxoid) for 16–18 h and turbidity adjusted to 0.5 McFarland Standard (optical density of 0.1 at wavelength of 625 nm) and stored at 4 °C. The bacterial suspension was further diluted 1:100 to achieve a working suspension of 10^6^ CFU/mL. The extract and the fractions were dissolved in 5.0% DMSO to achieve varying concentrations (1.563 to 50.0 mg/mL). Each of the concentration was impregnated into sterile 6 mm Whatman No. 1 filter paper disc. The disc was placed on Mueller Hinton Agar seeded with the test organism and incubated for 24 h at 37 °C. The diameter of inhibition of the test organism was recorded and the zone of inhibition was calculated according to the method of Aderiye and David [29].

### Statistical analysis

Data were presented as mean ± Standard Deviation of triplicate determination. Statistical analysis was done using SPSS (version 17) to analysis of variance (ANOVA) and Dunn’s Multiple Comparisons Test. The level of significance was set at 0.05.

## Results

**Fig. 1:**
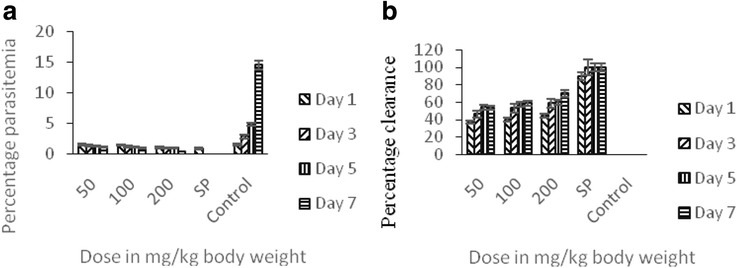
The prophylactic potentials of methanol extract of the stem bark of *T. orientalis* on *P. berghei*-induced malaria in mice showing the percentage parasitemia (**a**) and percentage clearance (**b**)

Figure 1 showed the prophylactic effects of the methanol fraction on the percentage parasitemia (Fig. 1a) and percentage clearance (Fig. 1b). The percentage parasitemia decreased as the dose increased while the parasite clearance increased as the dose increased. Percentage parasitemia of the treated animals in 200 mg/kg body weight group decreased significantly from day 3 to seven when compared with the untreated control. Again, the percentage parasite clearance increased significantly in the animals under the same dosage group when compared with the untreated control.

**Fig. 2:**
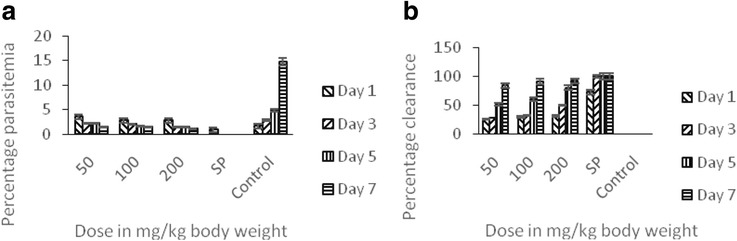
The prophylactic potentials of dichloromethane fraction of the methanol stem bark extract of *T. orientalis* on *P.berghei*-induced malaria in mice showing the percentage parasitemia (**a**) and percentage clearance (**b**)

In Fig. 2, the efficacy of the dichloromethane fraction (DCMF) of the methanol extract of *T. orientalis* as a chemopreventive drug candidates for malaria infection was presented. The figure representing the percentage parasitemia (Fig. 2a) showed that the parasite count decreased as the dose increased with the least parasitemia obtained at the highest dose administered. The results obtained also showed that all the doses used in this work compared favourably with SP with 200 mg being the dose that has the highest percentage clearance (Fig. 2b).

**Fig. 3:**
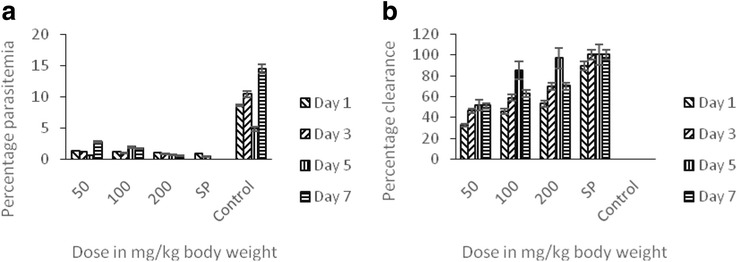
The prophylactic potentials of ethylacetate fraction of the methanol stem bark extract of *T. orientalis* on *P.berghei*-induced malaria in mice showing the percentage parasitemia (**a**) and percentage clearance (**b**)

In Fig. 3, it was shown that the ethylacetate fraction had the least efficacy as it had the highest percentage parasitemia even in the highest dose used (Fig. 3a) and the least parasite clearance as depicted in Fig. 3b.

**Fig. 4:**
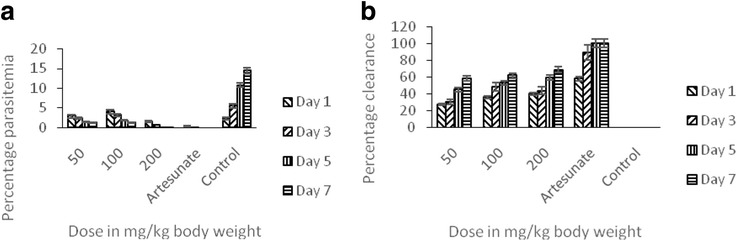
The prophylactic potentials of methanol fraction of the methanol stem bark extract of *T. orientalis* on *P.berghei*-induced malaria in mice showing the percentage parasitemia (**a**) and percentage clearance (**b**)

The chemopreventive effects of the methanol fraction as a potential prophylactic regimen for the prevention of malaria infection were presented in Fig. 4. The results obtained showed that the percentage parasitemia decreased (Fig. 4a) but zero parasitemia was not obtained when compared with SP. The percentage parasite clearance also was about 70% at the highest dose used (Fig. 4b)

Table 1 below explained that, at the lowest dosage of the extract (50 mg), the packed cell volume, white blood cell, neutrophil and lymphocyte of the extract were significantly higher than the control. Dichloromethane showed the highest value in these parameters having a packed cell volume of 40, white blood cell count of 7.95, neutrophil of 35 and lymphocyte of 60.5, which is an indication that it is more effective than the extract and other fractions. SP however shows a higher value in these parameters than dichlomethane.

**Table 1:** The hematological parameters of *P. berghei*-infected mice pretreated with various extracts of *T. orientalis*

Dose (mg/body weight)	Extract	PCV %	WBC%	Neutrophil	Lymphocyte	Monocyte	Eosinophil	Basophil
50	MEOH FR	30	6.00	33	53.5	1.5	-	-
	DCM	40	7.95	35	60.5	01	0.5	-
	MEOH EX	33	6.65	34	58.5	0.5	-	-
	ETOAC FR	28.5	5.35	30	50	01	-	-
	SP	43	6.4	34.5	65	-	-	-
	VEHICLE	30	5.6	32	48	-	-	-
100	MEOH FR	32.5	7.25	35	55.5	02	01	0.5
	DCM	41	8.00	36.5	65			
	MEOH EX	35	7.35	36	59	-	-	-
	ETOAC FR	30	6.8	34	53	-	-	-
	SP	43	6.4	34.5	65	-	-	-
	VEHICLE	30	5.6	32	48	-	-	-
200	MEOH FR	35	7.4	35.5	59	01	-	-
	DCM	42	10.2	38	67	01	-	-
	MEOH EX	38	8.00	36	61			
	ETOAC FR	32.5	7.8	35	55	-	-	-
	SP	43	6.4	34.5	65	-	-	-
	VEHICLE	30	5.6	32	48	-	-	-

KEY: *MEOH FR* Methanol Fraction, *DCMFR* Dichloromethane Fraction, *MEOH EX* Methanol Extract, *ETOAC FR* Ethylacetate Fraction, *SP* Sulfadoxine-pyrimethamine

At 100 mg dosage of the extract, the packed cell volume, white blood cell, neutrophil and lymphocyte values of the extract were significantly higher than the control. Dichloromethane showed the highest value in these parameters having a packed cell volume of 41, white blood cell count of 8, neutrophil of 35 and lymphocyte of 55.5, which is an indication that it is more effective than the other drug candidates. These values are higher in comparison to the lowest dosage of the extract as explained in Table 1 therefore; the extract and fractions showed a higher level of effectiveness as the dosage increased.

At the highest dosage of the extract and fractions, the hematological parameters including packed cell volume, white blood cell, neutrophil and lymphocyte presented values that were considerably higher than the control whereas dichloromethane showed the highest values of 42, 10.2, 38 and 67 in each of them respectively. This therefore is an indication that dichloromethane is more effective than the other drug candidates at the highest dosage. Again, SP showed a higher value in these parameters than dichlomethane.

**Fig. 5:**
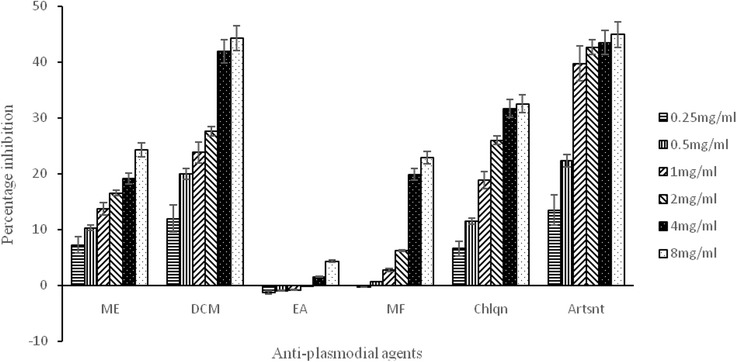
Percentage inhibition of β-hematin formation between extracts and fractions of *T. orientalis* and the standard controls. Key: ME = Methanol Extract, CM = Dichloromethane fraction, EA = Ethylacetate fraction, MF = Methanol Fraction, Chlqn = Chloroquine, Artsnt = Artesunate

The results of the in vitro antiplasmodial activity of the extract and fractions of *T. orientalis* are shown in Fig. 5 in comparison to two positive controls (both chloroquine and artesunate). The absorbance of the samples (400 nm) is inversely proportional to drug efficacy; the lower the absorbance, the more efficient the drug is. Although, all the drug candidates inhibited β-hematin formation, it is quite interesting to note that the dichloromethane fraction is the most potent fraction and is more effective than chloroquine phosphate.

As shown in Table 2; the extract and fractions of *T. orientalis* showed a concentration dependent antibacterial activity; meaning that the activity was higher at the highest concentration than at lower concentrations. In this antibacterial assay, methanol extract had a pronounced inhibitory effect on *Enterobacter cloaca* ATCC 13047 and *Enterococcus faecalis* ATCC 29212 while methanol fraction had the best inhibitory effects on *Escherichia coli* ATCC 8739 and *Serratiamercescens* ATCC 9986. The extract and the fractions affected both Gram-positive and Gram-negative bacteria alike thus, the susceptibility of the isolates to the extract and the fractions is as follows; *Serratiamercescens* ATCC 9986 < *Pseudomonas aeruginosa* ATCC 19582 < *Escherichia coli* ATCC 8739 < *Enterococcus faecalis* ATCC 29212 < *Enterobacter cloaca* ATCC 13047 < *Staphylococcus aureus* ATCC 6538. All the drug candidates screened performed lower than the Control 10 μg/mL gentamicin. Methanol extract had the highest inhibitory effect on *Enterobacter cloaca* followed by methanol fraction while ethylacetate fraction had the least effects on the isolate. The antibacterial activity of the extract and the fractions had no significant difference when compared at P˂0.05.

**Table 2:** Antibacterial activity of the extract and fractions of the methanol stem bark extract of *T. orientalis* on different bacterial isolates

Test Organisms	Extract/Fraction	Concentrations (mg/mL)					
		50.000	25.000	12.500	6.250	3.125	1.563
*Enterobacter cloaca* ATCC 13047	Ethylacetate fraction	35.54 ± 8.34	18.62 ± 3.54	0.00	0.00	0.00	0.00
	Dichloromethane fraction	58.24 ± 12.40	38.50 ± 11.74	3.14 ± 1.01	0.00	0.00	0.00
	Methanol Extract	102.14 ± 23.45	54.29 ± 10.81	7.07 ± 1.45	3.14 ± 1.32	0.00	0.00
	Methanol Fraction	48.69 ± 6.01	50.29 ± 9.33	12.57 ± 4.30	0.00	0.00	0.00
	Gentamicin (10 μg/mL)	211.13 ± 9.25					
*Escherichia coli* ATCC 8739	Ethylacetate fraction	50.29 ± 12.37	56.30 ± 11.80	28.29 ± 3.72	7.07 ± 3.23	0.00	0.00
	Dichloromethane fraction	66.24 ± 8.26	48.19 ± 9.83	32.73 ± 2.92	22.29 ± 1.56	0.00	0.00
	Methanol Extract	52.21 ± 9.36	35.50 ± 10.10	28.40 ± 3.30	12.57 ± 3.84	3.14 ± 0.44	0.00
	Methanol Fraction	71.53 ± 32.23	44.92 ± 8.54	19.14 ± 1.95	19.34 ± 0.85	3.14 ± 1.93	0.00
	Gentamicin (10 μ/ml)	144.52 ± 3.87					
*Serratiamercescens *ATCC 9986	Ethylacetate fraction	93.07 ± 6.12	73.12 ± 5.27	78.57 ± 16.34	7.07 ± 2.56	0.00	0.00
	Dichloromethane fraction	142.09 ± 15.28	95.07 ± 7.23	78.57 ± 10.67	52.21 ± 3.83	12.57 ± 3.45	3.14 ± 0.45
	Methanol Extract	63.6410.46±	63.64 ± 9.34	0.00	0.00	0.00	0.00
	Methanol Fraction	79.57 ± 8.10	96.07 ± 15.28	63.64 ± 3.67	61.45 ± 6.44	0.00	0.00
	Gentamicin (10 μ/ml)	111.13 ± 3.16					
*Pseudomonas aeruginosa* ATCC 19582	Ethylacetate fraction	64.31 ± 4.19	58.64 ± 7.28	53.29 ± 5.23	19.64 ± 2.45	3.83 ± 0.45	0.00
	Dichloromethane fraction	65.07 ± 24.34	28.29 ± 6.26	3.14 ± 0.34	0.00	0.00	0.00
	Methanol Extract	73.57 ± 12.33	50.29 ± 9.28	38.50 ± 11.36	4.56 ± 1.04	0.00	0.00
	Methanol Fraction	113.14 ± 23.12	63.64 ± 7.29	19.645.23	3.14 ± 0.77	0.00	0.00
	Gentamicin (10 μg/mL)	356.50 ± 12.07					
*Enterococcus faecalis* ATCC 29212	Ethylacetate fraction	122.79 ± 13.60	50.29 ± 13.92	19.64	3.36 ± 1.35	3.19 ± 0.31	0.00
	Dichloromethane fraction	38.50 ± 7.27	32.50 ± 4.65	12.57	0.00	0.00	0.00
	Methanol Extract	81.81 ± 5.73	28.29 ± 7.12	19.64	3.14 ± 0.25	0.00	0.00
	Methanol Fraction	52.19 ± 7.45	38.50 ± 3.71	38.50	0.00	0.00	0.00
	Gentamicin (10 μg/mL)	165.20 ± 14.02					
*Staphylococcus aureus* ATCC 6538	Ethylacetate fraction	52.19 ± 9.15	28.29 ± 3.27	29.29 ± 3.18	19.84 ± 3.23	3.18 ± 0.31	3.11 ± 0.23
	Dichloromethane fraction	53.82 ± 9.38	24.23 ± 3.55	0.00	0.00	0.00	0.00
	Methanol Extract	50.29 ± 18.39	28.49 ± 7.23	19.64 ± 3.91	0.00	0.00	0.00
	Methanol Fraction	53.23 ± 7.14	0.00	0.00	0.00	0.00	0.00
	Gentamicin (10 μg/mL)	50.29 ± 4.45					

## Discussion

The quest for cost effective and efficacious antimalarial drugs has made search for antiplasmodial drugs unending. The use of plant-based medicines for the treatment and prevention of malaria remain important in rural areas in Nigeria and probably other parts of Africa. This is partly because of the high cost of the patented drugs and unavailability of orthodox medicine in rural areas. In some areas in Africa, it is believed that herbs work better than the orthodox medicine. As a result of this, many herbs of plausible antimalarial properties had been authenticated and documented for their efficacy. *T. orientalis* is used as an antimalarial herb locally in Nigeria but this herb decoction and others used for the prevention of malaria purposes are usually taking daily and without a specific regimen. In folkloric medicine, such herbs that are usually taking as antimalarial prophylactics are *Enantia chloranta, Alstonia boonei*, etc. Apart from Nigeria, in Zimbabwe for example, local dwellers rely mostly on some herbs such as *Momordica balsamina L.*(leaf)*, Momordica foetida Schumach* (leaf) *and Adeniacis sampeloides* (Planch exHook) Harms (root) which are all used mainly as prophylactic regimen for malaria [30].

We are of the opinion that if such preparations worked without adverse effects, it is far better than therapeutic approach after an established infection of malaria. However, in areas where these herbs are taking either for treatment or prophylactic purposes, dose preparation is always a problem because herbal preparation is always taking ad libitum*.* This habit however can predispose the patients to the toxic effects of the secondary metabolites from the herbs.

From the results presented, it could be seen that the DCMF fraction had a good prophylactic effects in preventing malarial infection. This is because it suppressed the invasion of the Red Blood Cells (RBC) by the malaria parasites. Olanlokun [31] and his coworkers had shown that the DCMF fraction of this plant had the highest therapeutic antimalarial properties. It is quite interesting to note also, that this same fraction had the best chemopreventive properties against malaria parasite invasion. The GC-MS analysis of this fraction had shown the presence of fatty acid methyl esters. Several plant derived antimalarial agents have been identified. Alkaloids [32, 33] and non alkaloid natural products such as terpenes [34, 35], flavonoids, chromones, xanthones, anthraquinones and related compounds [36–41] had been well documented. However, miscellaneous compounds such as cyclic alkyl polyol derivatives and benzoic acid derivatives have also been shown to exhibit a relatively good antiplasmodial activity [42].

Innate immunity is an ancient and highly conserved system that provides a first line of defense against infection [43]. Immune cells such as monocyte/macrophages, natural killer (NK) cells, neutrophils, mast cells, dendritic cells (DCs), and certain T cell subsets, as well as epithelial and endothelial cells, sense the presence of pathogens or the tissue damage they cause, and respond by elaborating chemical signals (cytokines and chemokines). These pro-inflammatory mediators recruit and activate immune cells to destroy pathogens using various effector mechanisms, such as phagocytic clearance, production of reactive oxygen and nitrogen species, and secretion of anti-microbial peptides.

Phagocytosis of parasitized erythrocytes (PEs) and merozoites by myeloid cells is thought to be a primary mechanism for parasite clearance in non-immune individuals. Monocyte/macrophages and neutrophils have been shown to phagocytose PEs in vitro in the absence of malaria-specific antibodies [44, 45]. Other innate immune components contribute to control of malaria infection, such as PE destruction by platelets [46]. Parasites are also sensitive to oxidative stress: administration of pro-oxidants reduced parasitemia in *Plasmodium vinckei* infection [47]. Whether phagocyte production of reactive oxygen species (ROS) and nitric oxide (NO) contributes to control of parasite replication is controversial. Phagocyte-derived ROS and NO were shown to kill parasites in vitro [48, 49] and ex vivo neutrophil ROS production inversely correlated with parasite clearance time in Gabonese children [50]. If an increase in these blood cells minimize or at best control malaria parasite infection then, an increase in the production of these blood cells may be beneficial in defense mechanism. It is therefore not surprising that neutrophils, lymphocytes, monocytes, eosinophils and basophils increased in the group treated with DCM fraction of *T. orientalis.* Leucopenia was frequently seen in the malaria-infected animals that were not treated which was confirmed by other studies [51]. Anemia is one of the most common complications in malaria infection. This is because the infected blood cell membrane ruptured thus leading to hemolytic anemia. Low Packed Cell Volume (PCV) equally causes low hemoglobin content. Treatment with DCM fraction significantly increased the PCV and hemoglobin content compared with the untreated control and other drug candidates.

The intra-erythrocytic stage of malaria parasite is very important and is highly fundamental to the life cycle of this organism. This is because massive degradation of hemoglobin after a blood meal results in the liberation of monomeric heme which is highly toxic to the parasite. In order to survive, this parasite, as other blood parasites, have evolved a unique mechanism for heme detoxification through its conversion into an unreactive, nontoxic, insoluble crystalline pigment, known as hemozoin [52]. Disruption of this crystallization process results in the accumulation of this toxic molecule leading to the generation of Reactive Oxygen Species (ROS) which may induce oxidative stress consequently leading to the death of the parasite. Thus, this important stage serves as a target for action of several known antimalarial drugs [53, 54]. Also it is an excellent target for biochemistry studies and drug design in such a way that any drug candidate that can inhibit the formation of β-hematin can induce heme toxicity in the organism and so, parasite death occurs.

The in vitro antiplasmodial activity of the extract and fractions of *T. orientalis* showed that the DCM fraction had the highest inhibitory effect on β-hematin formation. The results obtained also showed that the DCM fraction compared favourably with artesunate and inhibit β-hematin formation better than chloroquine of the same concentration. The ability of this fraction to inhibit β-hematin formation significantly higher than chloroquine is highly appreciated while the ethylacetate fraction had the least activity. In the same vein, the extracts at high concentrations have inhibitory effects on both Gram-negative and Gram-positive bacteria.

Different parts of plant materials have been reported to exhibit anti-bacterial activities [55–57] Antibacterial compounds like octacosanoic acid, 1-octacosanyl acetate, simiarenone, simiarenol, episimiarenol and trematol have been isolated from stem bark of the plants [58]. Methanol extract had a pronounced inhibitory effect on the test isolates. This supported the report of Uddin [59] who reported ethanol extract of *T. orientalis* to be effective against both Gram-negative and Gram-positive bacteria. The activity of the plant may be due to the phytochemical constituents of the plant which justify its ethno medicinal usage among the rural dwellers [60].

From this study, it can be seen that various solvent preparations of the stem bark of *T. orientalis* were effective against malaria parasite thus, supporting the antiplasmodial indigenous claim of this plant. An improvement on the decoction preparation will be beneficial because most antimalarial herbs are locally extracted with water or other aqueous media.

## Conclusion

We hereby conclude that this work further substantiate the folkloric antimalarial and antibacterial use of this plant scientifically. In addition, we will suggest that the extraction procedures and solvent used be reviewed because the dichloromethane fraction was the most potent and locally, aqueous extraction is employed. It was again showed that the probable mechanism for the antiplasmodial effect was revealed to be via the heme metabolism in the malaria parasite.

## Abbreviations

ANOVA: Analysis of variance
ARDS: Acute respiratory distress syndrome
CFU: Colony forming unit
DCMF: Dichloromethane fraction
DCMP and DCMC: Dichloromethane Fraction Percentage Parasitemia and Clearance
DCs: Dendritic Cells
DMSO: Dimethylsulphoxide
EAP and EAC: Ethylacetate Fraction Percentage Parasitemia and Clearance
EF: Ethylacetate fraction
FePPIX: Ferriprotoporphyrin IX
ME: Methanol extract
MEP and MEC: Methanol Extract Percentage Parasitemia and Clearance
MF: Methanol fraction
MFP and MFC: Methanol Fraction Percentage Parasitemia and Clearance
NK: Natural Killer
NO: Nitric Oxide
*P. falciparum*: *Plasmodium falciparum*
*P.berghei*: *Plasmodium berghei*
PCV: Packed Cell Volume
PEs: Parasitized Erythrocytes
ROS: Reactive Oxygen Species
SDS: Sodiumduodecylsulphate
SP: Sulfadoxine-pyrimethamine
*T. orientalis*: *Trema orientalis*
UHAE: University Herbarium, Ado-Ekiti
VLC: Vacuum Liquid Chromatography
WBC: White Blood Cell
